# Micro RNA-148a Targets Bcl-2 in Patients with Non-Small Cell Lung Cancer

**DOI:** 10.31557/APJCP.2021.22.6.1949

**Published:** 2021-06

**Authors:** Ghada Nabil Elnaggar, Niveen M El-Hifnawi, Abeer Ismail, Maha Yahia, Reham A A Elshimy

**Affiliations:** 1 *Department of Clinical Pathology, National Cancer Institute, Cairo University, Egypt. *; 2 *Department of Medical Oncology, National Cancer Institute, Cairo University, Egypt.*

**Keywords:** MiR-148a- Bcl-2- NSCLC

## Abstract

**Objective:**

Lung cancer is one of the most prevalent cancers and the leading cause of cancer-related deaths worldwide. MicroRNAs regulate more than 60% of human genes, including tumor suppressor genes and oncogenes. Accordingly, they can affect cancer risk. This study aimed to evaluate the role of serum miR-148a as a non-invasive biomarker in non-small cell lung cancer (NSCLC) patients and to assess the correlation between miR-148a and Bcl-2, as one of its target proteins.

**Materials and Methods:**

A total of 50 newly diagnosed NSCLC cases and 30 apparently healthy controls were recruited in this study. MiR-148a level was measured by TaqMan- Real time RT-PCR assay and Bcl-2 level was measured by ELISA.

**Results:**

Significant lower expression of serum *miR-148a* and higher *serum Bcl-2* levels were observed in NSCLC patients as compared to the control group (p <0.001 each). A statistically significant inverse correlation was also evident between miR-148a and Bcl-2. Lower *miR-148a *gene expression level and higher Bcl-2 concentration were found to be associated with advanced tumor stage, lymph node involvement and distant metastasis.

**Conclusion:**

MiR-148a could be a possible biomarker for NSCLC and by targeting Bcl-2, it may offer a novel approach for treatment.

## Introduction

Lung cancer is a common malignant tumor and is considered a major cause of deaths related to cancer worldwide. (Wang et al., 2019). There are two main histological groups for lung cancer: non-small cell lung cancer (NSCLC) representing 85%, and 15% small cell lung cancer (SCLC) (Siegel et al., 2015).

Early detection is important in management of NSCLC. Several protein biomarkers have been used as diagnostic tools for this disease, such as CYFRA 21-1, CEA, TPS, SCC Ag and CA-125, but due to their limited sensitivity and specificity in early detection, it is significantly urgent to develop novel non-invasive biomarkers with high accuracy, as the five-year survival rate is about 80% in early stages, but drops sharply to approximately 14% in advanced stages (stage III/IV) (Geng et al., 2014).

MicroRNAs (miRNAs) are short non-coding endogenous RNA molecules that contain approximately 22 nucleotides. They function as key regulators in various biological processes and their dysregulation results in many diseases including cancer and autoimmune disorders (Liu et al., 2018).

MicroRNA-148a is a member of MiR-148/152 family located on chromosome 7p15.2. It is expressed normally in various human tissues including cerebral, heart, liver, thymus, pancreas, renal, placenta, uterus, testis, and the hematopoietic system (Li et al., 2016). It is a tumor suppressor gene and is found to be down-regulated in many types of human cancer. Evasion of apoptosis is a hallmark of cancers and is a common cause of therapeutic resistance (Sharma et al., 2019).

B cell lymphoma 2 (Bcl-2) is a critical molecule for regulating the apoptotic pathway (Youle et al., 2008). Bcl-2 signaling pathway plays important roles in human cancers. The activation of Bcl-2 has been shown to enhance tumor growth, invasion, motility, tumor spreading, metastasis, and inhibition of apoptosis (Adams and Cory, 2007). 

Several reports described the regulation of Bcl-2 by miR-148a and identified it as one of its target proteins in colorectal cancer (Zhang et al., 2011), pancreatic cancer (Zhang et al., 2014) and breast cancer (Li et al., 2017). 

This study aims to evaluate the role of serum miR-148a as a non-invasive biomarker in a group of NSCLC patients. We also aimed to study the correlation between serum level of Bcl-2 protein and its regulator miR-148a, and also their association with some clinicopathological parameters.

## Materials and Methods

*Patients*

This study was performed on a total number of 80 participants, including 50 newly diagnosed NSCLC cases at different disease stages, and 30 age-and sex-matched apparently healthy individuals as controls. Patients were recruited from the outpatient clinics at National Cancer Institute (NCI) hospital, Cairo University, from September 2016 till September 2017. The study was permitted by the Institutional Review Board (IRB) of the NCI, Cairo University. It was permitted according to the Helsinki guidelines of studies performed on human beings and a written consent was obtained from all study subjects before enrollment in the study. All patients were subjected to; full history taking and clinical examination, ordinary biochemical and hematological investigations, imaging techniques in the form of: chest X-rays, CT scan and MRI. All patients were either cytologically or histologically confirmed NSCLC cases. Samples were obtained from all patients prior to any therapeutic or surgical intervention. Participants’ age showed a mean ± SD of (58.1 ± 9.1) years in NSCLC cases and (57.7 ± 9.3) years in the control group. Patients’ characteristics are presented in table (1).

*Sample Collection*

Five milliliters of venous blood were withdrawn into 2 serum vacutainer tubes under complete aseptic precautions, allowed to clot for 30 minutes and centrifuged at 4000 R.P.M for 10 minutes. Yielded serum was divided into 2 micro tubes and stored at -80ºC till the time of analysis. First tube was used for serum quantification of miR-148a by qRT-PCR (Li et al., 2015). The second tube was used for determination of serum concentration of Bcl-2 by ELISA (Tas et al., 2005), and for determination of serum concentration of CEA by Cobas e411 Autoanalyzer, Roche. 

*Detection of serum miR-148a by Real-Time RT-PCR*

Total RNA, including miR, was extracted using the miRNeasy Mini Kit catalog#: 217004 (Qiagen, Germany), according to the manufacturer’s instructions.

Five µL of extracted total RNA were used as a template for synthesis of cDNA performed using TaqMan^® ^MicroRNA Reverse Transcription Kit (cat. no. 4366596) and Taqman MicroRNA Assay Primers manufactured by Thermo Scientific, USA. As recommended in the assay instructions provided, 15 µL of reverse transcription reaction components were used as a total volume. Each 15-µl reaction consists of 7 µl master mix, 3 µl of 5x primer, and 5 µl RNA sample.

The expression of *miR-148a *(gene of interest) was measured by qRT-PCR using TaqMan microRNA assay kit and TaqMan^® ^Universal PCR Master Mix II kit catalog No. 4440043 (Applied Biosystems). We pipetted 10 µl of TaqMan^®^ Universal PCR Master Mix II (2×), 1 µl of gene-specific TaqMan primer primers/ probe mix, and 6.5 µl of nuclear-free water. Then 2.5µl of each cDNA were pipetted in the corresponding well to reach a final volume of 20 µl per well. The subsequent reaction conditions were as follows: 95°C for 10 minutes then 45 cycles at 95°C for 15 seconds followed by 60°C for 1 minute.

TaqMan qRT-PCR was performed in duplicate, and U6 snRNA was used as endogenous reference for normalizing the expression level of *miR-148a* (Li et al., 2015). The relative* miR-148a *gene expression level was calculated using the equation 2^- ΔΔCT^ where ΔΔCT= (CT miR-148a - CT U6) patient sample - (CT miR-148a - CT U6) control sample (Livak and Schmittgen, 2001).

*Detection of serum protein Bcl-2 by ELISA technique*

Bcl-2 in serum was measured using sandwich Enzyme-linked Immunosorbent Assay Kit (catalog#: BMS244/3) for in vitro quantitative measurement of Bcl-2 supplied by eBioscience Corp. (Thermo Fisher Scientific, USA) according to the manufacturer’s instructions.

*Statistical Methods*

In this study, data was tested using SPSS version 25 (SPSS Inc., Chicago, IL, USA). Numerical parametric data was expressed as mean ± standard deviation (SD) while numerical non-parametric variables were expressed as median and range. Qualitative variables were expressed as frequency and percentage. Student T test was used to assess the statistical significance of the difference between two study groups means. For the comparison of more than two groups’ means, one-way analysis of variance (ANOVA) was used. To examine the relation between qualitative variables, Chi-square test or Fisher’s exact test was used. For comparing two different groups, the Mann Whitney U nonparametric test was used, while Kruskal-Wallis nonparametric test was used for more than two independent variables. To find a correlation between two variables Spearman’s rho (r) was calculated. The Receiver Operating Characteristic (ROC) curve was used to determine the cut-off values and to analyze the diagnostic utility of different markers. A p-value of less than 0.05 was considered statistically significant. All p-values are two sided.

## Results

*Down-regulation of serum miR-148a in NSCLC serum samples*

**Figure 1:**
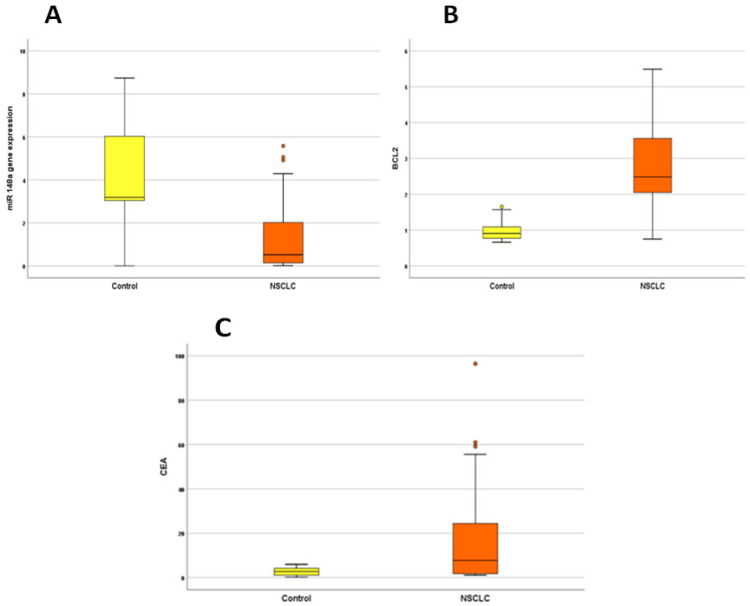
Box Plots for miR-148a, Bcl-2 and CEA Concentrations in NSCLC Cases versus Control. MiR-148a gene expression level is significantly lower in NSCLC group (A) compared to control group (p<0.001). Significantly higher Bcl-2 concentration (B) and CEA level (C) in NSCLC cases compared to control group (p<0.001 each).

MiR-148a was significantly down-regulated while Bcl-2 and CEA were up-regulated in the serum of NSCLC patients as shown in Figure 1 and Table 2.

*Receiver Operating Characteristic (ROC) Curve*

**Figure 2:**
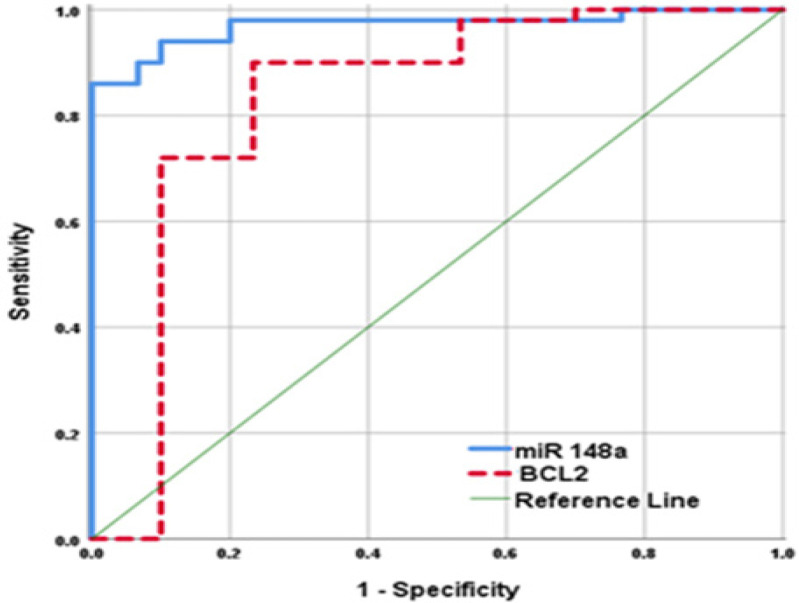
ROC of Serum miR-148a Gene Expression and Bcl-2 Concentration for Prediction of NSCLC Occurrence

The diagnostic value of miR-148a and Bcl-2 were evaluated by ROC analysis. The results shown in Figure (2), suggested that NSCLC patients could be distinguished from apparently healthy controls according to *miR-148a *expression at a cut-off value 3. The area under the curve (AUC) was 0.970 (95% CI 0.935-1, p<0.001), sensitivity was 90%, specificity was 93.3%, positive predictive value (PPV) was 95.7%, negative predictive value (NPV) was 84.8% and total accuracy was 91.2%. For Bcl-2, at a cut-off value 1.7 ng/ml, AUC was 0.829 (95% CI 0.718-0.941, p<0.001), sensitivity was 90%, specificity was 76.7%, PPV was 86.6%, NPV was 82.1% and total accuracy was 85%.

*Correlations of miR-148a and Bcl-2 in the studied groups*

Using Spearman’s correlation coefficient showed that significant negative correlation was found between miR-148a and Bcl-2, also there was a significant negative correlation between miR-148a with CEA, tumor size and stage. Bcl-2 showed a significant positive correlation with CEA, tumor size and stage as shown in Table 3.

*Relation between Serum miR-148a Level, Bcl-2 Level and Clinicopathological Characteristics of NSCLC Group*

The relative expression of serum *miR-148a *and *Bcl-2 *of the NSCLC patients were studied in relation to their clinicopathological data. MiR-148a was significantly lower in sera of patients with late stage (III and IV) than in those with early-stage NSCLC (p<0.001). Also, miR-148a had a statistically significant association with lymph node involvement (p<0.001) and with distant metastasis (p=0.002). Bcl-2 had a statistically significant association with stages (p<0.001), lymph node involvement (p=0.002) and distant metastasis (p<0.001). No significant differences were found with gender, smoking status, pathological subtypes, grade or differentiation as shown in Table 1.

**Table 1:** Association between Study Markers with Different Clinicopathological Characteristics in NSCLC Patients

	Variable	n	%	Serum miR-148a median (range)	P value	Serum Bcl-2 median (range)	P value
Gender	Male	44	88	0.5 (0.01 - 5.6)	0.56	2.5 (0.8 – 5.5)	0.87
	Female	6	12	0.7 (0.02 - 4.3)		2.4 (2.0 – 3.8)	
Smoking Status	Yes	44	88	0.5 (0.01 – 5.6)	0.56	2.5 (0.8 – 5.5)	0.87
	No	6	12	0.7 (0.02 – 4.3)		2.4 (2.0 – 3.8)	
Pathological Subtypes	Adenocarcinoma	26	52	0.4 (0.01 – 4.3)	0.095	2.8 (1.5 – 5.5)	0.085
	SCC	16	32	1.1 (0.01 – 5.6)		2.2 (0.8 – 4.6)	
	Large Undifferentiated	8	16	0.3 (0.01 – 2.2)		2.6 (2.2 – 5.1)	
Grade	I	3	6	2.8 (0.33 – 4.3)	0.353	2.1 (2.1 – 3.8)	0.725
	II	25	50	0.5 (0.01 – 5.6)		2.5 (0.8 – 5.5)	
	III	22	44	0.5 (0.01 – 5.1)		2.5 (1.2 – 5.1)	
Differentiation	Well	3	6	2.8 (0.33 – 4.3)	0.358	2.1 (2.1 – 3.8)	0.725
	Moderate	24	48	0.4 (0.01 – 5.6)		2.5 (0.8 – 5.5)	
	Poor	23	46	0.6 (0.01 – 5.1)		2.5 (1.2 – 5.1)	
Stage	I	12	24	2.9 (0.82 – 5.6)	<0.001*	2.0 (0.8 – 2.1)	<0.001*
	II	6	12	2.0 (0.74 – 2.3)		1.7 (1.5 – 2.3)	
	III	25	50	0.3 (0.01 – 2.2)		2.7 (2.3 – 3.8)	
	IV	7	14	0.1 (0.01 – 0.2)		4.3 (4.0 – 5.5	
	Early stage (I + II)	18	36	2.3 (0.7 – 5.6)	<0.001*	1.9 (0.8 – 5.5)	<0.001*
	Late stage (III + IV)	32	64	0.2 (0.01 – 2.2)		3.2 (2.3 – 5.5)	
Lymph Node Metastasis	Present	34	68	1.7 (0.05 – 5.6)	<0.001*	2.1 (0.8 – 3.8)	0.002*
	Absent	16	32	0.3 (0.01 – 2.3)		2.6 (1.5 – 5.5)	
Distant Metastasis	Present	6	12	0.7 (0.01 – 5.6)	0.002*	2.4 (0.8 – 4.3)	<0.001*
	Absent	44	88	0.1 (0.01 – 0.2)		4.4 (4.0 – 5.5)	

Qualitative data are represented as frequency (percentage); *P value <0.05 is significant; SCC, Squamous cell carcinoma.

**Table 2:** Level of miR-148a, Bcl-2 and CEA in NSCLC Patients and Control Group

Variable	NSCLC group (n=50)	Control group (n=30)	P value
Serum miR-148a	0.5 (0.01 - 5.6)	3.2 (0.004 – 8.7)	<0.001*
Serum Bcl-2	2.5 (0.8 – 5.5)	0.9 (0.7 – 1.7)	<0.001*
Serum CEA	7.7 (1.1 – 96.4)	2.7 (0.25 – 6)	<0.001*

*P value <0.05 is significant; Data shown as median (range).

**Table 3:** Correlations of miR-148a and Bcl-2 with Other Studied Parameters in NSCLC Cases

	miR-148a		Bcl-2	
	*R*	*P*	*R*	*P*
Age	-0.19	0.141	0.251	0.079
CEA	-0.279	0.049*	0.361	0.010*
Grade	-0.119	0.41	0.112	0.44
Differentiation	-0.083	0.568	0.094	0.518
Size	-0.456	0.001*	0.376	0.007*
Stage	-0.823	<0.001*	0.895	<0.001*
Bcl-2	-0.799	<0.001*	-	-

*P value <0.05 is significant.

## Discussion

Recently, the emergence of small non coding RNAs called microRNAs, that play important role in oncogenesis, has opened novel opportunities for early cancer diagnosis. MiRNAs have been found to regulate different cellular processes, such as proliferation, differentiation, cell cycle and apoptosis (Bartel, 2018). They have been associated with tumorigenesis by functioning as tumor suppressors or oncogenes (Debnath et al., 2017). There is a strong need to elucidate new mechanisms of NSCLC development, so as to establish an accurate and cost-effective screening method for this cancer. 

In this study, we evaluated the expression of serum *miR-148a* in 50 NSCLC patients and 30 controls. *MiR-148a *altered expression can be detected in various types of cancers and has been linked to the clinical classification and prognosis of tumors (Li et al., 2016).

Our data showed that miR-148a was significantly down-regulated in serum of NSCLC patients when compared to control group. This was in agreement with the studies of Li et al., (2015); Yang et al., (2015), Huang, (2016) and Kumar et al., (2020), proving that miR-148a is considered a tumor suppressor gene in lung cancer.

This result was also supported by several studies on tissue samples. Li et al., (2013), He and Xue (2017), Chen et al., (2017) and Bai et al., (2019) all reported that *miR-148a *expression was decreased in NSCLC tissues and cell lines compared with that in the corresponding adjacent normal lung tissues.

Similarly, the downregulated expression of *miR-148a *was reported in various types of cancers including gastric (Chen et al., 2013a; Zheng et al., 2014), colorectal (Takahashi et al., 2012), pancreatic (Li et al., 2016), liver (Heo et al., 2014), esophageal (Wijnhoven et al., 2010), breast (Li et al., 2017) and urogenital system cancers (Fujita et al., 2010; Zhou et al., 2012; Lombard et al., 2015).

MiR-148a regulates cell survival mechanisms by acting on different target genes to affect their function. As a tumor suppressor gene, it inhibits cell proliferation and induces apoptosis by suppressing the action of multiple proteins as Bcl-2 (Zheng et al., 2011; Wang, 2020), ROCK1 (Li et al., 2013), CCKBR (Zhang et al., 2014 a), and DNMT1 (Lombard et al., 2015). Thus, its down regulation is strongly associated with carcinogenesis.

In order to further investigate how miR-148a functions in lung cancer pathogenesis, Bcl-2 was studied as one of its molecular targets. We found that serum Bcl-2 concentration in NSCLC patients was significantly higher compared to the control group. This was in agreement with a study done by Tas et al., (2005) which supports that Bcl-2 is an important anti-apoptotic molecule, well accepted for NSCLC development (Huang et al., 2015).

Similarly, high Bcl-2 levels have been detected in a variety of tumor types, including small cell lung, melanoma, breast, prostate, colorectal, and bladder cancers, and especially in human lymphoid malignancies (Dai et al., 2016).

The results of the current study in serum samples are in keeping with data from the literature (Nalluri et al., 2015; D’Aguanno and Del Bufalo, 2020). These results suggest that decreased apoptosis associated with serum Bcl-2 elevation occur in lung cancer patients.

Regarding association between miR-148a with the clinicopathologic parameters examined in patients, our results revealed significant association between its low expression level with advanced tumor stage, lymph node involvement and distant metastasis. Also, miR-148a showed a significant negative correlation with CEA and tumor size. This can be referred to its role in the initiation and progression of NSCLC by acting on different target genes that inhibit cell migration and invasion as SMAD2 (Wang et al., 2013), DNMT1 (Lombard et al., 2015), MMP7 (Sakamoto et al., 2014), MET (Zhang et al., 2014b), and WNT1 (Yan et al., 2014). 

As for Bcl-2, there was also a significant association between its high level with advanced tumor stage, lymph node involvement and distant metastasis. A significant positive correlation was found between its level with CEA and tumor size. In addition, no significant correlation was found between either miR-148a or Bcl-2 with other clinicopathologic parameters including age, gender, smoking status, histological type, grade, or differentiation.

Others studies differ in what they demonstrated regarding the association between miR-148a or Bcl-2 with clinicopathological parameters in NSCLC patients. Huang (2016) compared the expression level of *miR-148a* between early-stage (stage I) and late-stage (stage II–III) NSCLC groups, and similar to our results, he found that the expression levels change significantly in correlation with the development of lung cancer. In concordance with results of the current study, Li et al., (2015) reported that low* miR-148a* expression was significantly correlated with presence of lymphatic metastasis. And similarly, no correlation was observed with other clinicopathologic factors, including age, gender, tumor differentiation, and histology. Also, Yang et al., (2015) reported significant association between *miR-148a* expression with tumor size and lymph node metastasis. Chen et al., (2013b) similarly reported that patients with lymph node metastasis and advanced clinical stage had a significantly lower expression of *miRNA 148a*.

Up to our knowledge, this is the first study that correlates serum Bcl-2 measured by ELISA with serum miR-148a in NSCLC. It revealed a significant inverse correlation between miR-148a and Bcl-2 in NSCLC and healthy groups with P-value <0.001 and correlation coefficient (r) = (-0.799).

Several studies described the regulation of Bcl-2 by miR-148a and identified it as one of its target proteins. This was reported in breast cancer by Li et al., (2017) who found that restoring expression of *miR-148a* suppressed the expression of *Bcl-2* at the level of both mRNA and protein, and also upregulation of miR-148a caused a subsequent reduction of proliferation and an increase in apoptosis, confirming their involvement in the oncogenesis of breast cancer.

Zhang et al., (2014a) similarly found that miR-148a regulates the growth and apoptosis in pancreatic cancer by targeting Bcl-2, and that the 3`-UTR of Bcl-2 is a functional target site for miR-148a silencing of Bcl-2.

Another study in colorectal cancer by Zhang et al., (2011) proved that miR-148a promotes apoptosis by targeting Bcl-2. And in agreement with our findings, a strong inverse correlation between them was observed (P-value <0.001 and correlation coefficient(r) = (-0.604).

The diagnostic value of miR-148a was evaluated by ROC analysis. AUC: 0.970 (95% CI: 0.935‒1) at Cut-off: 3, Sensitivity: 90%, Specificity: 93.3%. In agreement with our results, Li et al., (2015) reported that serum *miR-148a* gene expression level was significantly lower in NSCLC patients than that of healthy individuals. His study showed sensitivity of 77.8% and specificity of 80% at a cut-off level of 3.33. AUC was 0.775 (95% CI: 0.628‒0.885). Yang et al., (2015) also reported AUC: 0.90 (95% CI: 0.86‒0.95) with sensitivity of 85% and specificity of 83%.

Several studies were done to identify Bcl-2 inhibitors to be used for cancer therapy. Starting from the clinical use of antisense oligonucleotides directed against Bcl-2, and passing through BH3 mimetics that showed severe on-target toxicity, recent FDA approval of the BH3 mimetic Venetoclax validated the clinical relevance of using Bcl-2 anti-apoptotic members as therapeutic targets, not only for hematologic malignancies but also for breast carcinoma (D’Aguanno and Del Bufalo, 2020). We hope positive results can offer a way for these therapeutic strategies to be used for treatment of a large amount of solid malignancies including NSCLC.

In conclusion, miR-148a was down-regulated in NSCLC when compared to healthy controls which suggests that miR-148a may be used as a potential biomarker in NSCLC. Also, its expression was negatively correlated with serum *Bcl-2*, which was up-regulated in NSCLC patients, and this may explain the influence of miR-148a on increased risk of NSCLC and may offer a novel approach for treatment. Low *miR-148a *gene expression level was associated with advanced tumor stage, lymph node involvement and distant metastasis. So, it could be a prognostic factor in NSCLC.

## Author Contribution Statement

Niveen M El-Hifnawi, Abeer Ismail and Reham A.A. Elshimy planed and designed the research; Ghada Nabil Elnaggar participated in conception, interpretation of laboratory data and practical work. Maha Yahia and Ghada Nabil Elnaggar collecting clinical data and statistical analysis. All authors - contributing to the study design, participated in writing and editing the final version of the manuscript, read and approved the final manuscript.
